# Associations of minerals intake with colorectal cancer risk in the prostate, lung, colorectal, ovarian cancer screening trial

**DOI:** 10.3389/fnut.2024.1445369

**Published:** 2024-09-02

**Authors:** Siyue Li, Qingqian Ren, Zixuan Song, Baixue Liu, Dan Wang, Yanna Shang, Hao Wang

**Affiliations:** Department of Nutrition and Food Hygiene, College of Public Health, Zhengzhou University, Zhengzhou, China

**Keywords:** minerals, colorectal cancer, PLCO, zinc, iron, magnesium, copper, selenium

## Abstract

**Objective:**

Exploring the association between common mineral intake and the risk of colorectal cancer (CRC).

**Methods:**

We utilized the multivariate Cox proportional hazards model to assess the association between intake of minerals and the risk of CRC, estimating hazard ratios (HRs) and 95% confidence intervals (CIs).

**Results:**

A total of 101,686 eligible participants were included in the analysis of this study, including 1,100 CRC cases. After adjusting for potential confounders, we found that total zinc intake (HR_Q4vs.Q1_: 0.79, 95%CI 0.67–0.93; *P* for trend <0.05), iron intake (HR_Q4vs.Q1_: 0.81, 95%CI 0.68–0.96; *P* for trend <0.05), copper intake (HR_Q4vs.Q1_: 0.80, 95%CI 0.68–0.95; *P* for trend <0.05), selenium intake (HR_Q4vs.Q1_: 0.83, 95%CI 0.69–0.98; *P* for trend <0.05) were significantly negatively associated with the incidence of CRC, but magnesium intake in the appropriate range is associated with a reduced risk of CRC (HR_Q3vs.Q1_: 0.77, 95%CI 0.65–0.91; *P* for trend >0.05).

**Conclusion:**

Our findings suggested that an appropriate intake of total zinc, iron, copper, selenium and magnesium were associated with lower CRC risk.

## 1 Introduction

Minerals intake is strongly associated with cancer, especially digestive system tumors. In recent years, colorectal cancer (CRC) has become the third most commonly diagnosed cancer, and second leading cause of cancer death worldwide (1, 2). Such a high morbidity of CRC is not only associated with the promotion of screening programs, but is also closely linked to modern dietary habits (3). The incidence of CRC is tending to occur at progressively younger ages and the proportion of young patients (age < 50 years) is increasing (4, 5). The colorectum, a vital component of the digestive system, is increasingly burdened by the disease (1). CRC is a common cancer with a significant genetic component; approximately 10–16% of patients have pathogenic variants in their cancer susceptibility genes (6). In addition, lifestyle factors such as smoking, alcohol consumption, obesity, and the intake of red and processed meats also influence CRC risk (2, 7). Several studies have demonstrated the association of minerals with carcinogenesis and the content of certain minerals are significantly differences between tumor tissues and healthy tissues (8–10). The minerals are necessary in the metabolism of the body, but there are fewer studies on the relationship between mineral intake and CRC.

The minerals, specifically magnesium (Mg), zinc (Zn), iron (Fe), copper (Cu) and selenium (Se), can be acquired in diet or supplements. These minerals maintain normal physiological functions and are important for maintaining human health, such as in DNA replication, immunity and energy production (11–14). The association between minerals and cancers has aroused great concern. Increasing dietary zinc intake was reported to be effective in reducing cancer risk, especially in prostate cancer (15–17). Iron deficiency and iron deficiency anemia are global health problems, whereas excessive iron intake may be associated with tumorigenesis (18–21). Copper is involved in the important process of cancer development, with lower in malignant tumors than in benign tumors, and it was reported that copper of specific structure has anti-tumor effects (22–25). The anti-cancer effects of selenium have been confirmed by several studies (9, 26, 27). Through further research, it was noted that all of these minerals have been linked to CRC. Epidemiologic studies and some meta-analyses have shown higher intake of magnesium is associated with lower CRC risk (28, 29). Iron has also been found to reduce the risk of lung cancer (30), but it may increase the risk of CRC (29). Moreover, elesclomol-mediated copper overload has been shown to inhibit CRC both *in vitro* and *in vivo* (31). A randomized controlled trial has shown that higher selenium levels are associated with a lower risk of prostate, lung and CRC (32). Some researchers have found higher levels of magnesium, zinc, copper and selenium in tumor tissue from CRC patients than in normal tissue (8). Based on these finding, we explored the association between the intake of these elements and the development of CRC.

In recent years, the effect of minerals intake on CRC has become a subject of considerable concern, but study findings have varied considerably. The Prostate, Lung, Colorectal, and Ovarian (PLCO) cancer screening trial, a prospective cohort study, has a large number of participants, a long follow-up period and reliable mineral intake data, which can effectively show the relationship between the intake of each mineral and the development of CRC. Our study aims to examine the associations between the intake of five key minerals (magnesium, zinc, copper, iron, and selenium) and the CRC risk in the PLCO cancer screening trial.

## 2 Methods

### 2.1 Study population

The design and methodology of the PLCO cancer screening trial has been reported in several previous studies (33, 34). The PLCO Cancer Screening Trial is a randomized, controlled trial of screening tests for prostate, lung, colorectal and ovarian cancers and more than a dozen other cancers. Ten PLCO Screening Centers recruited approximately 155,000 participants aged between 55 and 75 years from November 1993 to July 2001, and all participants signed an informed consent. The Clinical Trials. gov numbers for PLCO are NCT00002540, NCT01696968, NCT01696981, and NCT01696994.

### 2.2 Data collection and minerals assessment

All participants were asked to complete a baseline questionnaire (BQ), is the baseline risk factor questionnaire, including participant-reported information such as sex, age, education, cancer history and medical history. The Dietary History Questionnaire (DHQ) is a food frequency questionnaire that was added in 1998 and covers daily intake of 124 foods over the past 12 months for 113,000 participants. Nutrient intake was derived from frequencies and portion sizes from the Food Frequency Questionnaire (FFQ), in which mineral values per portion multiplied by the frequency of daily intake and then summed to obtain the intake of the nutrient concerned (35). Daily intakes of nutrients were calculated based on the Nutrition Data System for Research (NDS-R). The NDS-R combines nutritional information from the USDA Standard Reference Nutrient Database, food manufacturers, scientific literature, and other published food tables (36). In this study, total intake of five minerals (magnesium, zinc, iron, copper, and selenium) was extracted from DHQ, both from food and from supplements.

### 2.3 Participant selection

**Figure 1:**
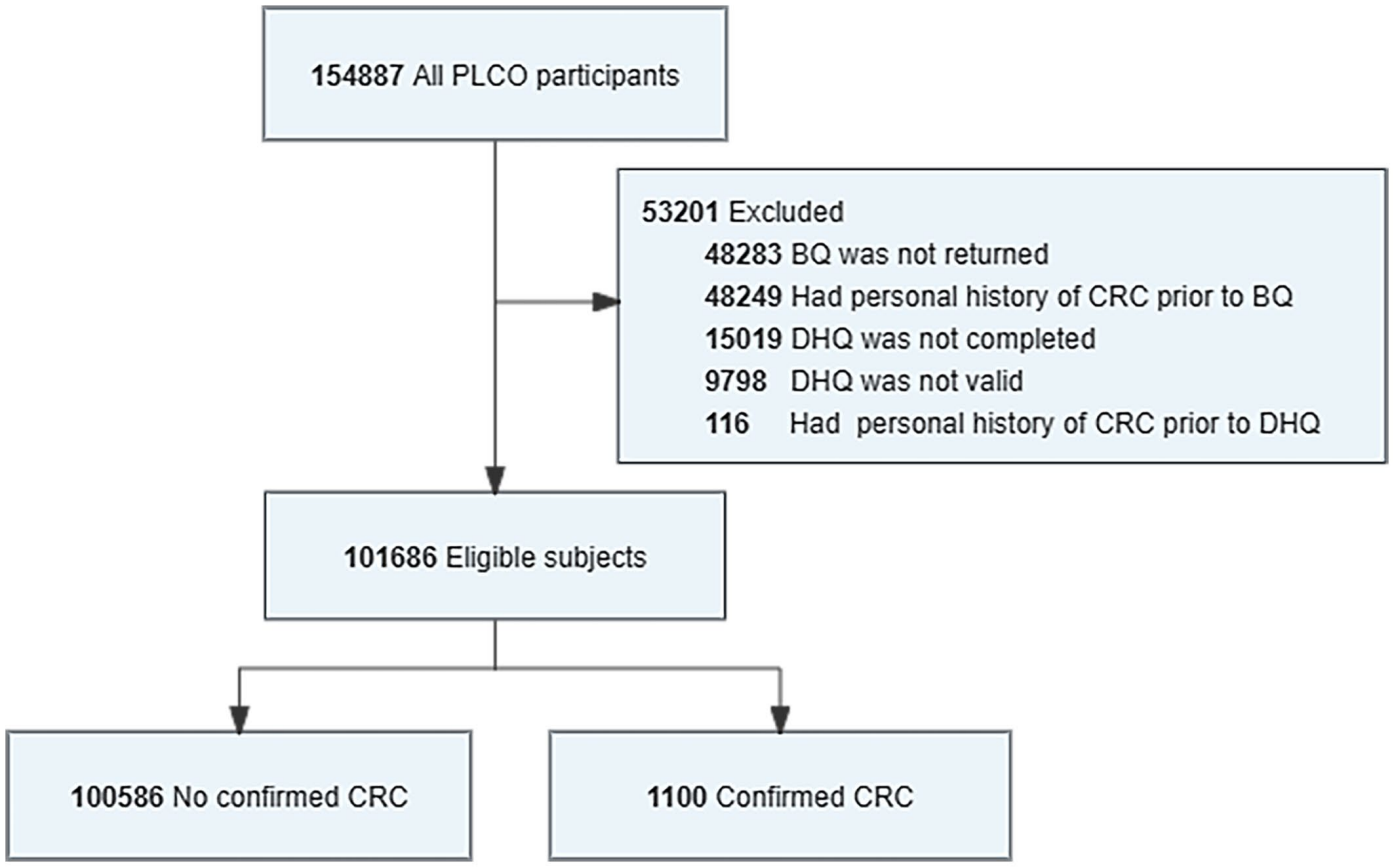
Flowchart for identifying eligible PLCO participants in study analysis. The final cohort had a total of 101,686 eligible participants included in the analysis. BQ, baseline questionnaire; DHQ, diet history questionnaire.

Our study needed to identify participants eligible for the DHQ CRC analysis (Figure 1). Participants will be excluded from the study if they did not return a baseline questionnaire (*n* = 48,283); missing DHQ completed data (*n* = 15,019); their DHQ was invalid (*n* = 9,798); they have personal history of any cancer prior to the DHQ (*n* = 116). After screening, 101,686 eligible participants were identified in the analysis, including 1,100 CRC cases.

### 2.4 Ascertainment of CRC

The endpoint event in this study was CRC incidence. Carcinoid colorectal cancer is considered a target for CRC screening, it is therefore included in the definition of confirmed CRC. In the PLCO trial, subjects were not diagnosed with CRC at the start of the study. CRC reports were collected in a variety of ways, including self-reports, family reports and death certificates, family reports and death certificates. Cancer data collected up to December 31, 2009 and mortality data collected through 2015 for each subject in the PLCO trial, and cancers and deaths continue to accrue. The time metric chosen for the study was the number of days between completion of the DHQ and the diagnosis for participants with CRC, or to trial exit otherwise.

### 2.5 Statistical analysis

Hazard ratios (HRs) values in this study were calculated using data after multiple interpolation. The distribution of general characteristics of cases and controls were compared using Chi-square (*χ*^2^) tests for categorical variables and *t*-test for continuous variables. Intakes of total magnesium, zinc, iron, copper, and selenium were used to generate new categorical variables by quartiles.

HRs and 95% confidence intervals (CIs) were estimated for CRC risk in relation to five minerals intake using a multivariate Cox proportional hazards model. Modelling to adjust covariates for known or suspected CRC risk factors, including age (<65 vs. ≥65), sex (male vs. female), randomization arm (intervention vs. control), body mass index (BMI, <25 kg/m^2^ vs. ≥25 kg/m^2^), race (White, Non-Hispanic vs. Other), marital status (married vs. unmarried), education (<college vs. ≥college), smoking status (never smoked cigarettes vs. current cigarette smoker vs. former cigarette smoker), drinking status (never vs. former vs. current), family history of CRC (yes vs. no vs. possibly-relative or cancer), and family history of any cancer (yes vs. no vs. possibly-relative or cancer). Three regression models were constructed for each of the five minerals: model 1 does not make any adjustments and roughly estimates HR; model 2 just adjusted for age and sex; model 3 adjusted for all ten covariates.

Subgroup analysis was done for 5 variables such as age, sex, BMI, family history of CRC and family history of any cancer. The results were corrected for 10 confounders other than the grouping factors. Restricted spline models were fitted with three nodes to determine dose-response trends between intakes of total minerals (continuous variable) and CRC risk. Sensitivity analyses were performed by excluding events with less than 2 years of follow-up, extreme BMI values (<1% and >99%), or removing cases with missing values.

The proportional hazards assumption was graphically tested for all built models, all data are consistent with the proportional risk assumption. Statistical analyses were performed using R statistical software (http://www.R-project.org, The R Foundation), and a *p* value <0.05 (two-tailed) was considered significant.

## 3 Result

### 3.1 Participant characteristics

The characteristics of the study population are shown in Table 1. The median follow-up time for cancer diagnosis data was 11.3 years, during which 1,100 cases of CRC were diagnosed. Mean age at baseline was 64.14 years in the case group and 62.38 years in the control group. By comparing the case and control groups, it can be seen that the case group’s characteristics included a majority of males, a younger age, higher BMI, a higher likelihood of having a higher education, primarily alcohol drinkers, and a family history of any cancer.

**Table 1:** Baseline characteristics of CRC patients and non-patient participants.

Variables	Overall (*n* = 101,686)	Non-patients (*n* = 100,586)	Patients (*n* = 1,100)	*p*
Age (years), mean (SD)	62.40 (5.28)	62.38 (5.28)	64.14 (5.19)	<0.001
Sex (*n*, %)				
Male	49,445 (48.63)	48,837 (48.55)	608 (55.27)	<0.001
Female	52,241 (51.37)	51,749 (51.45)	492 (44.73)	
Arm (*n*, %)				
Intervention	51,771 (50.91)	51,327 (51.03)	444 (40.36)	<0.001
Control	49,915 (49.09)	49,259 (48.97)	656 (59.64)	
Education (*n*, %)				
<college	42,911 (42.20)	42,387 (42.14)	524 (47.64)	0.001
>=college	58,578 (57.61)	58,005 (57.67)	573 (52.09)	
Missing	197 (0.19)	194 (0.19)	3 (0.27)	
Marital status (*n*, %)				
Married	98,286 (96.66)	78,733 (78.27)	851 (77.36)	0.306
Unmarried	3,214 (3.16)	21,671 (21.54)	245 (22.27)	
Missing	186 (0.18)	182 (0.18)	4 (0.36)	
Race (*n*, %)				
White, non-Hispanic	92,470 (90.94)	91,480 (90.95)	990 (90.00)	0.432
Other	9,179 (9.03)	9,069 (9.02)	110 (10.00)	
Missing	37 (0.04)	37 (0.04)	0 (0.00)	
BMI (*n*, %)				
<25	34,428 (33.86)	34,094 (33.90)	334 (30.36)	0.006
>=25	65,919 (64.83)	65,176 (64.80)	743 (67.55)	
Missing	1,339 (1.32)	1,316 (1.31)	23 (2.09)	
Smoking (*n*, %)				
Never	48,535 (47.73)	48,050 (47.77)	485 (44.09)	0.108
Former	43,744 (43.02)	43,237 (42.99)	507 (46.09)	
Current	9,394 (9.24)	9,286 (9.23)	108 (9.82)	
Missing	13 (0.01)	13 (0.01)	0 (0.00)	
Alcohol intake (*n*, %)				
Never	10,110 (9.94)	10,013 (9.95)	97 (8.82)	0.602
Former	14,746 (14.50)	14,581 (14.50)	165 (15.00)	
Current	73,950 (72.72)	73,141 (72.71)	809 (73.55)	
Missing	2,880 (2.83)	2,851 (2.83)	29 (2.64)	
Family history of CRC (*n*, %)				
No	88,118 (86.88)	87,190 (86.68)	928 (84.36)	0.040
Yes	10,301 (10.13)	10,178 (10.12)	123 (11.18)	
Possibly	2,493 (2.45)	2,453 (2.44)	40 (3.64)	
Missing	774 (0.76)	765 (0.76)	9 (0.82)	
Family history of any cancer (*n*, %)				
No	44,584 (43.84)	44,130 (43.87)	454 (41.27)	0.223
Yes	56,821 (55.88)	56,178 (55.85)	643 (58.45)	
Missing	281 (0.28)	278 (0.28)	3 (0.27)	

Values are means (SD) for continuous variables or number (percentage) for categorical variables. Differences in characteristics shown patients and non-patients were tested by using chi-square tests for categorical variables and ANOVA for continuous variables.

### 3.2 Association between intakes of total minerals and the incidence of CRC

Estimates risk of CRC associated with total intakes of magnesium, zinc, iron, copper, and selenium are shown in Table 2. Model 1 was the crude model; model 2 just adjusted for age and sex; model 3 adjusted for all ten covariates. There was a significant correlation between CRC incidence and moderate magnesium intake in the crude analysis model (HR_Q3vs.Q1_: 0.76, 95%CI: 0.64–0.91, *p* = 0.002). Similar results were found in the adjusted models (model 2: HR_Q3vs.Q1_: 0.75, 95%CI: 0.63–0.89, *p* = 0.001; model 3: HR_Q3vs.Q1_: 0.77, 95%CI: 0.65–0.91, *p* = 0.002). Zinc intake was found to significantly associated with lower CRC risk. After adjusting for covariates, the results of models 2 and 3 were the same as model 1. After adjusting for ten covariates, the minerals iron, copper, and selenium were also shown to associate with lower risk of CRC, *P* for trend <0.05.

**Table 2:** Association between minerals intake and CRC risk in the PLCO cancer screening trial.

Variables	Cohort (*n*)	Cases (*n*)	Person-years	Incidence rate per 10,000 person-years	HR (95% CI), *p*-value		
					Model 1	Model 2	Model 3
Magnesium (mg/day)							
Q1 (<273.64)	25,422	304	222512.0	13.66	Ref	Ref	Ref
Q2 (≥273.64 to <354.05)	25,423	282	224442.2	12.56	0.92 (0.78–1.08), *p* = 0.313	0.91 (0.78–1.07), *p* = 0.273	0.93 (0.79–1.10), *p* = 0.394
Q3 (≥352.05 to <446.42)	25,422	235	225394.4	10.43	0.76 (0.64–0.91), *p* = 0.002	0.75 (0.63–0.89), *p* = 0.001	0.77 (0.65–0.91), *p* = 0.002
Q4 (≥446.42)	25,419	279	224155.1	12.45	0.91 (0.77–1.07), *p* = 0.263	0.87 (0.74–1.02), *p* = 0.095	0.89 (0.75–1.05), *p* = 0.154
*P* for trend					0.142	0.041	0.072
Zinc (mg/day)							
Q1 (<9.93)	25,428	334	223224.8	14.96	Ref	Ref	Ref
Q2 (≥9.93 to <19.82)	25,444	254	224148.8	11.33	0.76 (0.64–0.89), *p* = 0.001	0.75 (0.64–0.88), *p* = 0.001	0.76 (0.64–0.89), *p* = 0.001
Q3 (≥19.82 to <25.75)	25,399	250	224807.3	11.12	0.74 (0.63–0.88), *p* < 0.001	0.77 (0.65–0.90), *p* = 0.001	0.79 (0.67–0.93), *p* = 0.004
Q4 (≥25.75)	25,415	262	224322.8	11.68	0.78 (0.66–0.92), *p* = 0.003	0.77 (0.65–0.90), *p* = 0.001	0.79 (0.67–0.93), *p* = 0.004
*P* for trend					0.004	0.004	0.011
Iron (mg/day)							
Q1 (<13.65)	25,436	311	222720.2	13.96	Ref	Ref	Ref
Q2 (≥13.65 to <24.03)	25,407	275	224777.9	12.23	0.88 (0.75–1.03), *p* = 0.111	0.85 (0.72–1.00), *p* = 0.053	0.87 (0.74–1.03), *p* = 0.097
Q3 (≥24.03 to <31.72)	25,444	265	224716.4	11.79	0.84 (0.72–1.00), *p* = 0.044	0.86 (0.73–1.02), *p* = 0.080	0.89 (0.76–1.05), *p* = 0.176
Q4 (≥31.72)	25,399	249	224289.3	11.10	0.80 (0.67–0.94), *p* = 0.007	0.78 (0.66–0.92), *p* = 0.003	0.81 (0.68–0.96), *p* = 0.014
*P* for trend					0.007	0.008	0.028
Copper (mg/day)							
Q1 (<1.23)	25,653	316	224803.5	14.06	Ref	Ref	Ref
Q2 (≥1.23 to <2.34)	25,242	268	223027.0	12.02	0.86 (0.73–1.01), *p* = 0.060	0.83 (0.71–0.98), *p* = 0.029	0.85 (0.72–1.00), *p* = 0.046
Q3 (≥2.34 to <3.2)	25,697	273	226958.9	12.03	0.86 (0.73–1.01), *p* = 0.060	0.88 (0.75–1.04), *p* = 0.138	0.91 (0.77–1.07), *p* = 0.251
Q4 (≥3.2)	25,094	243	221714.2	10.96	0.78 (0.66–0.92), *p* = 0.004	0.77 (0.65–0.91), *p* = 0.003	0.80 (0.68–0.95), *p* = 0.010
*P* for trend					0.008	0.015	0.047
Selenium (mcg/day)							
Q1 (<59.52)	25,434	306	222865.6	13.73	Ref	Ref	Ref
Q2 (≥59.52 to <81.59)	25,410	272	225181.8	12.08	0.88 (0.75–1.04), *p* = 0.125	0.87 (0.73–1.02), *p* = 0.086	0.88 (0.74–1.03), *p* = 0.114
Q3 (≥81.59 to <110.78)	25,429	251	224691.4	11.17	0.81 (0.69–0.96), *p* = 0.016	0.78 (0.66–0.92), *p* = 0.004	0.79 (0.66–0.93), *p* = 0.006
Q4 (≥110.78)	25,413	271	223764.9	12.11	0.88 (0.75–1.04), *p* = 0.134	0.82 (0.69–0.98), *p* = 0.028	0.83 (0.69–0.98), *p* = 0.029
*P* for trend					0.151	0.032	0.031

**Figure 2:**
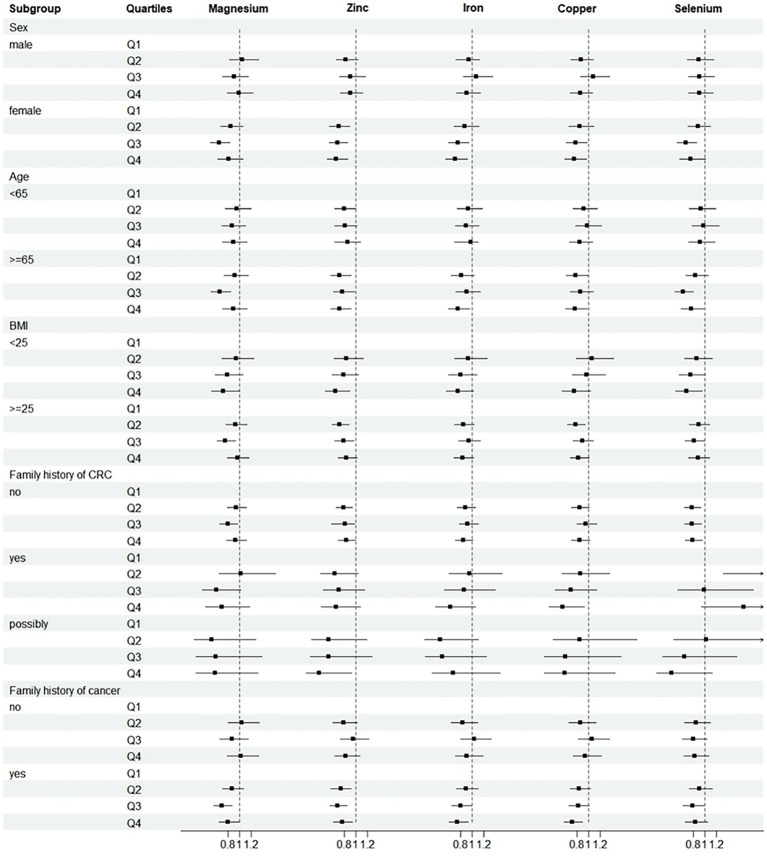
Subgroup analyses of CRC incidence. Adjusted for age (<65 vs. ≥65), sex (male vs. female), trial arm (intervention vs. control), race (White, non-Hispanic vs. other), marital status (married vs. unmarried), education (<college vs. ≥college), BMI (<25 kg/m^2^ vs. ≥25 kg/m^2^), smoking status (never vs. current vs. former), alcohol drinking status (never vs. current vs. former), family history of CRC (no vs. yes vs. possibly), family history of any cancer (no vs. yes). Subgroup analyses by sex, age, BMI, family history of CRC and family history of cancer. HRs were adjusted except for the stratification factor. *p* > 0.05 was considered no statistical significance.

**Figure 3:**
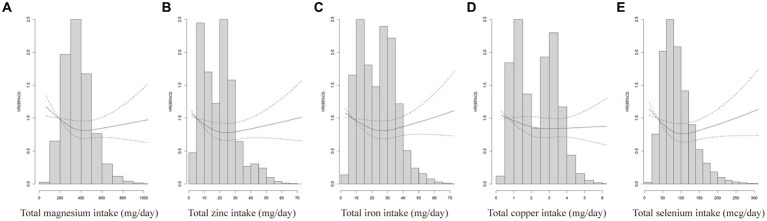
Does-response analyses for the associations between minerals intake and CRC incidence. Does-response analyses for the associations between total magnesium intake **(A)**, total zinc intake **(B)**, total iron intake **(C)** total copper intake **(D)** total selenium intake **(E)** and CRC. HRs and 95%CIs were calculated by the fully-adjusted multivariable Cox regression model, including age (<65 vs. ≥65), sex (male vs. female), trial arm (intervention vs. control), race (White, non-Hispanic vs. other), marital status (married vs. unmarried), education (<college vs. ≥college), BMI (<25 kg/m^2^ vs. ≥25 kg/m^2^), smoking status (never vs. current vs. former), alcohol drinking status (never vs. current vs. former), family history of CRC (no vs. yes vs. possibly), family history of any cancer (no vs. yes).

Further analysis was performed after adjusting for confounders. Subgroup analyses suggested that the protective effect of these five minerals against CRC was more pronounced among men, age ≥ 65 years, or with family history of any cancer (Figure 2). Sensitivity analyses showed that the results of the association between minerals intake and CRC risk were generally consistent with those in Table 2 after removing missing data, removing BMI extremes, or removing cases with less than 2 years of follow-up (Table 3). Restricted cubic spline model analysis suggested that there was a nonlinear association of Mg, Fe, Zn, and Se intakes with incidence of CRC (*P*-non-linear <0.05, Figures 3A–E).

**Table 3:** Sensitivity analyses on the association between minerals intake and CRC incidence.

Processing methods	Quartiles	HR (95% CI), *p*-value				
		Magnesium	Zinc	Iron	Copper	Selenium
Primary analysis	Q1	Ref	Ref	Ref	Ref	Ref
	Q2	0.93 (0.79–1.10), *p* = 0.394	0.76 (0.64–0.89), *p* = 0.001	0.87 (0.74–1.03), *p* = 0.097	0.85 (0.72–1.00), *p* = 0.046	0.88 (0.74–1.03), *p* = 0.114
	Q3	0.77 (0.65–0.91), *p* = 0.002	0.79 (0.67–0.93), *p* = 0.004	0.89 (0.76–1.05), *p* = 0.176	0.91 (0.77–1.07), *p* = 0.251	0.79 (0.66–0.93), *p* = 0.006
	Q4	0.89 (0.75–1.05), *p* = 0.154	0.79 (0.67–0.93), *p* = 0.004	0.81 (0.68–0.96), *p* = 0.014	0.80 (0.68–0.95), *p* = 0.010	0.83 (0.69–0.98), *p* = 0.029
	*P* for trend	0.072	0.011	0.028	0.047	0.031
Deleting missing values	Q1	Ref	Ref	Ref	Ref	Ref
	Q2	0.91 (0.77–1.08), *p* = 0.273	0.79 (0.67–0.94), *p* = 0.007	0.86 (0.73–1.02), *p* = 0.091	0.83 (0.70–0.98), *p* = 0.029	0.92 (0.77–1.08), *p* = 0.308
	Q3	0.75 (0.62–0.89), *p* = 0.001	0.79 (0.67–0.94), *p* = 0.008	0.88 (0.75–1.05), *p* = 0.159	0.91 (0.76–1.07), *p* = 0.245	0.79 (0.66–0.95), *p* = 0.011
	Q4	0.88 (0.74–1.04), *p* = 0.145	0.79 (0.66–0.93), *p* = 0.006	0.81 (0.68–0.96), *p* = 0.017	0.80 (0.67–0.95), *p* = 0.010	0.84 (0.70–1.01), *p* = 0.057
	*P* for trend	0.073	0.010	0.032	0.055	0.044
Excluding participants with extreme BMI	Q1	Ref	Ref	Ref	Ref	Ref
	Q2	0.95 (0.80–1.12), *p* = 0.526	0.77 (0.65–0.91), *p* = 0.002	0.88 (0.75–1.04), *p* = 0.131	0.87 (0.73–1.02), *p* = 0.088	0.88 (0.74–1.04), *p* = 0.127
	Q3	0.78 (0.66–0.93), *p* = 0.005	0.80 (0.67–0.94), *p* = 0.007	0.91 (0.77–1.07), *p* = 0.255	0.93 (0.79–1.09), *p* = 0.376	0.80 (0.67–0.94), *p* = 0.009
	Q4	0.90 (0.76–1.07), *p* = 0.236	0.79 (0.67–0.94), *p* = 0.006	0.82 (0.69–0.97), *p* = 0.022	0.81 (0.68–0.96), *p* = 0.016	0.84 (0.70–1.00), *p* = 0.045
	*P* for trend	0.061	0.016	0.045	0.066	0.050
Excluding participants with a follow-up less than 2 years	Q1	Ref	Ref	Ref	Ref	Ref
	Q2	0.87 (0.72–1.04), *p* = 0.134	0.77 (0.64–0.92), *p* = 0.005	0.86 (0.72–1.04), *p* = 0.113	0.82 (0.68–0.99), *p* = 0.038	0.88 (0.73–1.06), *p* = 0.174
	Q3	0.71 (0.58–0.86), *p* < 0.001	0.74 (0.61–0.89), *p* = 0.002	0.85 (0.71–1.03), *p* = 0.098	0.88 (0.74–1.06), *p* = 0.187	0.72 (0.59–0.87), *p* = 0.001
	Q4	0.81 (0.67–0.97), *p* = 0.024	0.76 (0.63–0.92), *p* = 0.004	0.77 (0.64–0.94), *p* = 0.008	0.73 (0.60–0.88), *p* = 0.001	0.78 (0.64–0.95), *p* = 0.012
	*P* for trend	0.011	0.005	0.012	0.009	0.008

## 4 Discussion

In this study, we analyzed the relationship between total minerals intake and CRC incidence and found that total minerals intake was associated with the risk of CRC incidence. However, there is not a simple negative or positive association between intake and risk of morbidity. According to our findings, the risk of CRC is at its lowest when the total intake of minerals is as follows: magnesium between 352.05 and 446.42 mg/day (Q3), zinc between 9.93 and 19.82 mg/day (Q2), iron exceeding 31.72 mg/day (Q4), copper exceeding 3.2 mg/day (Q4), and selenium between 81.59 and 110.78 mcg/day (Q3) (Table 2). These results have been adjusted for potential confounders.

Regarding the relationship between magnesium and zinc intake and CRC incidence, our findings are in agreement with some previous studies that higher magnesium and zinc intake are associated with a lower risk of CRC incidence. The Netherlands cohort study on diet and cancer has found that higher magnesium intake is more protective for people with a BMI > 25 (37). A cohort study in Japan found that higher dietary intake of magnesium may reduce the risk of CRC in Japanese men (38). The Swedish mammography cohort, a population based prospective cohort of women, showed that high magnesium intake reduces the incidence of CRC in women (39). The findings for zinc and CRC are similar to magnesium. The Iowa Women’s Health Study followed 34,708 postmenopausal women for 15 years. Based on this study, Lee et al. found that zinc intake was associated with a decreased risk of distal colon cancer (*P* for trend = 0.03) (40). Larsson et al., analyzing data from the population-based Swedish mammography cohort, proposed a relatively weak association of zinc intake with colon cancer (41). However, our study suggests that higher zinc intake is significantly associated with a reduced CRC risk. While these studies vary in population characteristics, the conclusions mostly suggest that magnesium and zinc play a protective role against CRC, and our findings contribute to the existing evidence on this topic. Distinct from the aforementioned studies, our data were derived from the PLCO database, the study population included both men and women, and with a long follow-up period and a large sample size, the results obtained were adjusted for multiple confounders.

In recent years, there have been relatively few studies on selenium and CRC. The selenium and vitamin E cancer prevention trial (SELECT) was a randomized, placebo-controlled trial of 35,533 men followed for a minimum of 7 years and a maximum of 12 years between August 22, 2001, and June 24, 2004. Lippman et al., analyzed data from this trial to assess the potential of selenium and vitamin E in preventing prostate cancer, with prespecified secondary outcomes including lung and CRC. But selenium was not statistically significantly associated with CRC (compared to the placebo group, selenium HR = 1.05 99% CI = 0.66–1.67) (42). However, our findings indicate that selenium is a protective factor against CRC when selenium intake is in the range of 81.59–110.78 mcg/day, with statistically significant results in three adjusted models. Comparing these findings with those of the above studies, we can hypothesize that the protective effect of selenium against CRC is more pronounced in women, which is confirmed in our results.

There are many differing views on the effect of iron intake on CRC. Summarizing past studies, we find that one American, one Canadian and one French case-control study all concluded that higher risks of CRC was observed for iron intake (43–45). But a case-control study from Australia suggested that iron has been observed to reduce the risk of CRC (46). All of the above are case-control studies. In addition, a European prospective cohort study finds that iron intake was not associated with CRC risk (HR_Q5vs.Q1_: 0.88; 95%CI: 0.73, 1.06) (47). After our analysis of the PLCO database, we found that the risk of CRC was significantly decreased when total iron intake reached the fourth quartile (*P* for trend <0.05). Comparing these studies, we hypothesize that the differing findings may be due to the proportion of dietary versus supplemental iron intake, a topic that necessitates further exploration. In addition, the different study population, length of follow-up, and sample size of the cohort study may have contributed to the differences in results from previous studies.

The association between copper intake and cancer has been widely reported, but the specific effect of copper intake on the risk of CRC in human populations remains unclear. Some studies have suggested that copper may reduce the risk of lung and esophageal cancer (48), while others have reported that there is no evidence of an association between dietary copper intake and cancer development (21, 49). The results of a case-control study in Burgundy, France showed the odds ratios associated with the fourth quartile of intake were 2.4 (95%CI, 1.3–4.6) (50). In contrast, by analyzing data from 1,100 CRC patients in the PLCO database, we found that copper may reduce cancer risk. The difference between the results of previous studies and our study may be due to the small number of cases, differences in the dietary structure of the population, and unavoidable recall bias.

### 4.1 Strengths and limitations of this study

Strengths of this study include its prospective design, a large population sample size, a long follow-up period, a high level of confidence in the authenticity and validity of the outcome screening, and very detailed information about the diet. However, this study has some limitations. Firstly, the sample population in this study was mostly non-Hispanic whites, which may affect the generalization of the findings to other populations. Secondly, residual confounders could not be completely excluded despite adjusting for some confounders using the three models. Lastly, this study did not assess the effects of the interaction of genetic factors and various minerals in the development of CRC.

## 5 Conclusion

In conclusion, for the analysis of PLCO, a large US cohort, our findings not only corroborate the conclusions of previous studies on the protective role of minerals magnesium and zinc in the development of CRC, but also suggest new perspectives on the role of copper, iron, and selenium in CRC. Minerals factors play an important role in the prevention of disease, and an in-depth study of minerals can be beneficial in the prevention of cancer.

## Data Availability

The data analyzed in this study is subject to the following licenses/restrictions: requires PLCO approval. Requests to access these datasets should be directed to https://cdas.cancer.gov/plco/.
